# Trends of fast food consumption among adolescent and young adult Saudi girls living in Riyadh

**DOI:** 10.3402/fnr.v59.26488

**Published:** 2015-03-18

**Authors:** Nora A. ALFaris, Jozaa Z. Al-Tamimi, Moneera O. Al-Jobair, Naseem M. Al-Shwaiyat

**Affiliations:** 1 Nutrition and Food Science Department, College of Home Economics, Princess Nourah Bint Abdulrahman University, Riyadh, Saudi Arabia; 2 Department of Clinical Nutrition, College of Applied Health Sciences in Arrass, Qassim University, Buraydah, Saudi Arabia

**Keywords:** fast food, restaurants, adolescent, young adult, Saudi Arabia

## Abstract

**Background:**

Saudi Arabia has passed through lifestyle changes toward unhealthy dietary patterns such as high fast food consumption. Adolescents and young adults, particularly girls, are the main groups exposed to and affected by these adverse eating behaviors.

**Objective:**

The aim of this study was to examine the trends of fast food consumption among adolescent and young adult Saudi girls living in Riyadh, and to compare between them.

**Design:**

In a cross-sectional survey, 127 adolescent Saudi girls (13–18 years) and 69 young adult Saudi girls (19–29 years) were randomly recruited to participate in this study. Weight, height, waist circumference, and hip circumference were measured using standardized methods. Twenty-four-hour diet recall and a face-to-face interview food questionnaire were performed.

**Results:**

Most of the participants had adequate intake of protein, riboflavin, iron, and sodium, but exhibited low intake for several other nutrients. Among study participants, 95.4% consume restaurants’ fast food and 79.1% eat fast food at least once weekly. Burgers and carbonated soft drinks were the main kinds of fast food meals and beverages usually eaten by girls. Adolescent girls who usually ate large portion sizes of fast food had significantly higher mean waist circumference and hip circumference. Participants eat fast food primarily for enjoying the delicious taste, followed by convenience. Restaurants’ hygiene and safety standards were the main concern regarding fast food for 62.2% of girls. Finally, international restaurants were preferable by participants to buy fast food compared with local restaurants (70.9% vs. 29.1%).

**Conclusion:**

Our findings provide evidence on the high prevalence of fast food consumption among Saudi girls, suggesting an urgent need for community-based nutrition interventions that consider the trends of fast food consumption and targeted eating behaviors of adolescent and young adult girls.

Adolescence is a crucial life stage characterized by dramatic modifications in lifestyle patterns. These modifications include more unhealthy food choices, eating outside the home (mainly at fast food restaurants), sedentary behaviors, and physical inactivity, especially among girls, all of which put adolescents at nutritional risk (1, 2). Many of teenagers’ dietary behaviors may be related to some distorted perceptions adopted by them. A study reported that adolescent girls associated consumption of fast food with pleasure, friends, and independence, while they associated consumption of healthy food with parents and being at home (3). Current data emphasized that environmental influences, especially family, had important effects on eating habits, weight gain, and physical activity during the transition from adolescence to adulthood (4, 5). When adolescents form certain dietary behaviors, they will maintain these behaviors even after becoming adults and establishing new households that are independent of their parents and family (5). Therefore, if healthful dietary behaviors are not well formed in adolescents and undesirable lifestyle patterns persisted during the transition to adulthood, these behaviors may carry out for a lifetime, which would increase the risk for chronic non-communicable diseases such as obesity 6–8) .

Fast food typically refers to food that is quickly prepared, purchased in self-service from restaurants with precooked ingredients, and served in a packaged form to the customer to take-away such as burgers, French fries, and pizza (9). Fast food first popularized in the 1970s in the United States, which has today the largest fast food industry in the world. American consumption of food prepared outside home increased from 18% within 1977–1978 to 32% within 1994–1996 of total energy. In addition, meals and snacks based on food prepared outside home contained more calories, and were higher in total fat and saturated fat and lower in dietary fiber, calcium, and iron, than home-made foods (10). Moreover, a strong positive association has been reported between fast food consumption and both weight gain and insulin resistance, suggesting that fast food increases the risks of obesity and type 2 diabetes (11).

Adolescents and young adults form the main consumers for fast food meals compared with older people (2, 12). As taste, time considerations, convenience, and cost are major factors that contribute to an adolescent's or young adult's food choices, fast food restaurants serve as popular sites for their meals eaten outside the home (13, 14). Fast food contains more fat, saturated fat, added sugars, added salt, and energy and less dietary fiber; therefore, eating fast food seems to have an adverse effect on diet quality (15–17). French et al. (18) investigated fast food consumption among 4,746 school students aged 11 to 18 years and reported that about 75% of adolescents ate at fast food restaurants during the week previous to the survey. Moreover, consuming fast food was associated with lower intakes of fruits, vegetables, and milk. In the same way, Morse and Driskell (19) examined the trends of fast food consumption among college students. Their results clarified that most young adults have reported eating meals at fast food restaurants 1–3 times weekly. In a 10-year longitudinal study, Schmidt et al. (20) examined the trends of fast food consumption and its relationship to diet quality among black and white adolescent girls. They found that fast food intake was positively associated with intake of energy, sodium, total fat, and saturated fat. In addition, the frequency of fast food consumption increased with age in both races.

Saudi culture is strongly religious, conservative, and family oriented. While women's status is high in the family, especially in the roles of mothers and sisters, women usually remain out of public view and contact only with their related men. In the public restaurants, women are required to use specially designated family sections. Consequently, for women, eating in restaurants means more than consuming a meal for hunger. It is an opportunity to go outside the home and gather with family or female friends. In addition, female students reported a higher prevalence of dieting, greater positive attitudes toward healthy eating, and greater interest in their health, body weight, and body image than their male counterparts. Consequently, female students are more likely to be respondents for nutrition education programs (21–23). Therefore, the purpose of this report was to study the trends of fast food consumption of a group of adolescent and young adult Saudi girls and to compare between them. It was based on two hypotheses. First, fast food consumption is common among Saudi girls. Second, adolescents and young adults may have differences in trends of fast food consumption as fast food eating may change with age.

## Methods

### Design and participants

The current study is a cross-sectional survey conducted during March–April, 2010 in Riyadh, the capital city of Saudi Arabia. All of the participants are Saudi girls, aged 13–29 years, school or college students, and living in Riyadh, and they were randomly selected. The adolescent girls (13–18 years) were recruited from the intermediate and secondary school complex in Princess Nourah Bint Abdulrahman University, whereas the young adult girls (19–29 years) were recruited from the campus of Princess Nourah Bint Abdulrahman University. One hundred and ninety-six girls agreed to participate in this study after obtaining a written consent in accordance with the Helsinki Declaration. The study was approved by the Nutrition and Food Science Department, College of Home Economics, Princess Nourah Bint Abdulrahman University, Riyadh, Saudi Arabia.

### Anthropometric measurements

Body weight was measured with minimal clothing and without shoes to the nearest 0.1 kg using a calibrated portable scale. Height was measured to the nearest 1 cm using a stadiometer, while the subject was in a full standing position without shoes. Body mass index (BMI) was calculated as the ratio of weight (kg) to height (m^2^). Waist circumference was measured at halfway between the lower border of the ribs and the iliac crest in a horizontal plane. Hip circumference was measured at the widest point over the buttocks. Both waist and hip circumferences were measured using a non-stretchable tape to the nearest 1 cm. The waist–hip ratio was calculated as the ratio of waist circumference to hip circumference.

### Dietary data collection

Habitual nutrients intake was assessed using 24 h diet recall for the previous day. A nutrient analysis software program was used (Food Processor for Windows, version 7.71, ESHA Research, Salem, OR, USA) to estimate the daily intake of several nutrients for each participant in the form of the nutrient adequacy ratio (NAR), which is the ratio of actual nutrient intake to the recommended intake of that nutrient based on dietary reference intakes (DRIs) (24).

A descriptive food questionnaire was designed by the researchers to study the trends of fast food consumption. Face validity for the questionnaire was assessed by using a pilot-tested group of 20 participants from the target population to ensure that the questions are understandable. A face-to-face interview questionnaire was performed with the respondents after defining fast food and giving them an overview about the study. The socio-demographic variables were collected from participants, which included age group, family size and income, and parents’ educational level. The questionnaire investigated the trends of fast food consumption by using 16 items, divided into three parts; fast food consumption pattern (7 items), attitude toward fast food (5 items), and fast food restaurant use (4 items).

### Data analysis

The Statistical Package for Social Sciences (SPSS Inc., Chicago, IL, USA) version 21 was used for data analysis. Categorical variables were expressed as numbers and percentages, and analyzed using a chi-square test. Continuous variables were expressed as means and standard deviations, and analyzed using a one-way ANOVA test. All reported *p* values were made on the basis of two-tailed tests. Differences were considered statistically significant at *p*<0.05.

## Results

### Socio-demographic and anthropometric characteristics

This study includes 196 Saudi girls; 64.8% of them were adolescent school students aged 13–18 years, and the rest of them were young adult college students aged 19–29 years (Table 1). Most of the participants have a large family size consisting of at least six members, and high family income exceeds 2,000 USD. Furthermore, 43.4% of mothers and 67.9% of fathers earned a college education or higher. However, there are no significant differences between both age groups regarding any of the above-mentioned characteristics. The mean BMI of young adult girls (23.6) was higher than that of adolescent girls (22.4) but not significantly. However, mean waist circumference and hip circumference were significantly higher among young adult girls compared to adolescent girls.

**Table 1:** The socio-demographic and anthropometric characteristics of participants

Variables	Adolescents (13–18 years)	Young adults (19–29 years)	Total (13–29 years)	*p*
Age group	127 (100%)	69 (100%)	196 (100%)	
Family size^a^				
5 members or less	22 (17.3%)	15 (21.7%)	37 (18.9%)	0.450
6 members or more	105 (82.7%)	54 (78.3%)	159 (81.1%)	
Family income^a^				
2,000 USD or less	38 (29.9%)	21 (30.4%)	59 (30.1%)	1.000
More than 2,000 USD	89 (70.1%)	48 (69.6%)	137 (69.9%)	
Mother's education level^a^				
Secondary school education or less	72 (56.7%)	39 (56.5%)	111 (56.6%)	0.706
College education or higher	55 (43.3%)	30 (43.5%)	85 (43.4%)	
Father's education level^a^				
Secondary school education or less	42 (33.1%)	21 (30.4%)	63 (32.1%)	0.982
College education or higher	85 (66.9%)	48 (69.6%)	133 (67.9%)	
Anthropometric measurements^b^				
Height (cm)	157.9 (6.5)	159.5 (6.0)	158.4 (6.4)	0.085
Weight (kg)	55.7 (11.3)	60.0 (11.9)	57.2 (11.7)	0.013
Body mass index (kg/m^2^)	22.4 (4.4)	23.6 (4.6)	22.8 (4.5)	0.074
Waist circumference (cm)	73.1 (11.7)	78.9 (13.4)	75.1 (12.6)	0.002
Hip circumference (cm)	96.3 (14.2)	101.5 (18.2)	98.1 (15.9)	0.029
Waist–hip ratio	0.77 (0.11)	0.78 (0.10)	0.77 (0.11)	0.236

a Categorical variables were expressed as numbers and percentages, and analyzed using a chi-square test.

b Continuous variables were expressed as means and standard deviations, and analyzed using a one-way ANOVA test.

### Nutrients intake

The mean NAR of protein was 1.84 and 1.58 for adolescent and young adult girls, respectively, which indicates that protein intake met the dietary requirement in most subjects (Table 2). Riboflavin was the only vitamin for which most of the participants achieved adequate intake (mean NAR was 2.41). Similarly, most girls’ intake of iron, sodium, and phosphorus (for adolescent girls only) was adequate. However, most girls exhibit low intake of several other vitamins and minerals, especially pantothenic acid, biotin, folic acid, vitamin D, vitamin E, selenium, and manganese, as mean NAR was lower than 1.0. Moreover, mean NARs of vitamin C, calcium, phosphorus, potassium, and sodium were significantly higher among adolescent girls compared to young adult girls.

**Table 2:** Participants’ nutrient adequacy ratio (NAR) of several nutrients

Nutrients	Adolescents, mean (SD)	Young adults, mean (SD)	Total, mean (SD)	*p* a
Protein	1.84 (1.40)	1.58 (1.05)	1.75 (1.29)	0.192
Thiamin	0.68 (1.15)	0.42 (0.45)	0.59 (0.97)	0.071
Riboflavin	2.56 (3.72)	2.14 (3.34)	2.41 (3.59)	0.427
Niacin	0.72 (1.80)	0.40 (0.65)	0.61 (1.50)	0.152
Pantothenic acid	0.04 (0.09)	0.06 (0.14)	0.05 (0.11)	0.355
Biotin	0.02 (0.04)	0.03 (0.11)	0.02 (0.07)	0.229
Folic acid	0.03 (0.08)	0.03 (0.08)	0.03 (0.08)	0.797
Vitamin B6	0.16 (0.65)	0.09 (0.22)	0.13 (0.54)	0.399
Vitamin B12	0.27 (0.46)	0.35 (0.73)	0.30 (0.58)	0.328
Vitamin C	0.33 (0.53)	0.17 (0.31)	0.27 (0.47)	0.029
Vitamin A	0.45 (0.49)	0.39 (0.39)	0.43 (0.46)	0.449
Vitamin D	0.0004 (0.004)	0.01 (0.03)	0.002 (0.02)	0.078
Vitamin E	0.02 (0.04)	0.02 (0.04)	0.02 (0.04)	0.947
Iron	1.21 (1.10)	1.04 (0.98)	1.14 (1.06)	0.289
Calcium	0.55 (0.74)	0.28 (0.21)	0.46 (0.62)	0.003
Phosphorus	1.72 (3.37)	0.74 (0.63)	1.37 (2.77)	0.018
Potassium	0.71 (1.61)	0.27 (0.27)	0.55 (1.32)	0.024
Magnesium	0.33 (1.09)	0.16 (0.26)	0.27 (0.89)	0.211
Zinc	0.18 (0.39)	0.18 (0.35)	0.18 (0.37)	0.877
Sodium	2.18 (3.82)	1.18 (1.01)	1.83 (3.17)	0.035
Selenium	0.05 (0.30)	0.02 (0.11)	0.04 (0.25)	0.436
Manganese	0.02 (0.10)	0.05 (0.12)	0.03 (0.10)	0.210

a Continuous variables were expressed as means and standard deviations, and analyzed using a one-way ANOVA test.

### Fast food consumption pattern

**Fig. 1:**
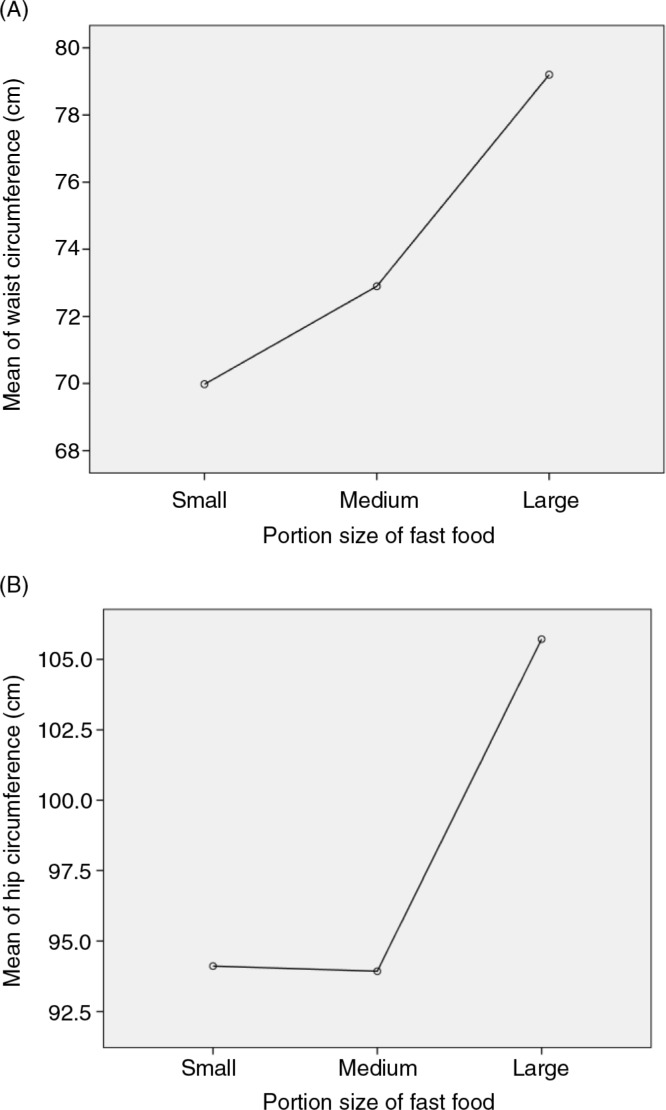
This means plots diagram illustrates the relationship between the mean of waist circumference (A) and mean of hip circumference (B) of adolescent girls and usually ordered portion size of fast food.

Results showed that the vast majority of the participants (95.4%) eat restaurant fast food (Table 3). Fast food was consumed once per week by 52.8% of adolescent girls and 60.9% of young adult girls. Moreover, 25.2% of adolescent girls and 20.3% of young adult girls consumed fast food twice or more weekly. In total, 79.1% of the sample eats fast food at least once weekly. Beef or chicken burgers were the main kinds of fast food meals usually eaten by the sample subjects (70.4%), followed by pizza (32.7%) and French fries (29.6%). A significantly higher rate of young adult than adolescent girls reported eating pizza usually (49.3% vs. 23.6%). On the other hand, only 4.1% of the participants usually consume hotdog. Regarding portion size, most of the participants usually ordered either small (37.2%) or medium (44.9%) portion sizes of fast food meals. However, the large portion size was the choice for 17.9% of participants. Adolescent girls who usually eat the large portion size of fast food had significantly higher mean waist circumference (*p*=0.006) and mean hip circumference (*p*=0.001) (Fig. 1). Interestingly, carbonated soft drinks were the main beverages usually consumed with fast food meals by both adolescent and young adult girls (89 and 75.4%, respectively), but other beverages such as coffee, tea, sweetened fruit drinks, or energy drinks were rarely consumed with fast food. Finally, weekends and evenings were the frequent times when the participants often consumed fast food meals.

**Table 3:** Participants’ responses for part 1 of the fast food questionnaire (regarding fast food consumption patterns)

Questions asked and answer levels	Adolescents, *N* (%)	Young adults, *N* (%)	Total, *N* (%)	*p* a
1. Do you eat restaurants fast food regularly?				
A. Yes	121 (95.3%)	66 (95.7%)	187 (95.4%)	0.904
B. No/sometimes	6 (4.7%)	3 (4.3%)	9 (4.6%)	
2. How often do you consume fast food?				
A. Once per month or less	28 (22%)	13 (18.8%)	41 (20.9%)	0.547
B. Once per week	67 (52.8%)	42 (60.9%)	109 (55.6%)	
C. Twice per week or more	32 (25.2%)	14 (20.3%)	46 (23.5%)	
3. Do you usually eat each of the following fast foods? (Yes or no)				
A. Beef or chicken burger (yes)	93 (73.2%)	45 (65.2%)	138 (70.4%)	0.241
B. Pizza (yes)	30 (23.6%)	34 (49.3%)	64 (32.7%)	<0.001
C. French fries (yes)	33 (26%)	25 (36.2%)	58 (29.6%)	0.133
D. Hotdog (yes)	5 (3.9%)	3 (4.3%)	8 (4.1%)	0.890
E. Doughnuts (yes)	13 (10.2%)	14 (20.3%)	27 (13.8%)	0.051
F. Croissant (yes)	9 (7.1%)	7 (10.1%)	16 (8.2%)	0.455
4. Which portion size of fast food do you usually eat?				
A. Small (regular)	44 (34.6%)	29 (42%)	73 (37.2%)	0.502
B. Medium	58 (45.7%)	30 (43.5%)	88 (44.9%)	
C. Large	25 (19.7%)	10 (14.5%)	35 (17.9%)	
5. What kind of beverages do you usually drink with fast food?				
A. Carbonated soft drinks	113 (89%)	52 (75.4%)	165 (84.2%)	0.167
B. Coffee or tea	1 (0.8%)	2 (2.9%)	3 (1.5%)	
C. Fresh fruit juices	10 (7.9%)	12 (17.4%)	22 (11.2%)	
D. Sweetened fruit drinks	2 (1.6%)	2 (2.9%)	4 (2%)	
E. Energy drinks	1 (0.8%)	1 (1.4%)	2 (1%)	
6. When do you usually consume fast food during the week?				
A. Beginning of the week	1 (0.8%)	1 (1.4%)	2 (1%)	0.890
B. Middle of the week	6 (4.7%)	3 (4.3%)	9 (4.6%)	
C. Weekend	76 (59.8%)	38 (55.1%)	114 (58.2%)	
D. No specific time	44 (34.6%)	27 (39.1%)	71 (36.2%)	
7. When do you usually consume fast food during the day?				
A. Morning	0 (0%)	2 (2.9%)	2 (1%)	0.179
B. Afternoon	2 (1.6%)	2 (2.9%)	4 (2%)	
C. Evening	83 (65.4%)	39 (56.5%)	122 (62.2%)	
D. No specific time	42 (33.1%)	26 (37.7%)	68 (34.7%)	

a Categorical variables were expressed as numbers and percentages, and analyzed using a chi-square test.

### Attitude toward fast food

As mentioned in Table 4, the participants eat fast food primarily for enjoying the delicious taste, followed by convenience. Adolescent girls had greater interest in taste compared with young adult girls (55.9% vs. 42%). In contrast, young adult girls were more concerned about convenience than adolescent girls (34.8% vs. 19.7%). Restaurants’ hygiene and safety standards were the main concern regarding fast food for 62.2% of girls, and the restaurant's location was the main concern for another 19.9% of them. As expected, most participants evaluated the taste of fast food as excellent (80.6%) and the price of fast food as acceptable (82.7%). A significantly higher percentage of adolescent girls (85.8%) than young adult girls (71%) found that fast food meals are of excellent taste. Surprisingly, more than half of the girls (53.1%) believed that fast food has either high or acceptable nutritional value. In addition, 27.6% of participants had no idea about the nutritional value of fast food. Finally, a higher rate of young adult girls (27.5%) than adolescent girls (15%) believed that fast food has low nutritional value.

**Table 4:** Participants’ response for part 2 of the fast food questionnaire (regarding attitude toward fast food)

Questions asked and answer levels	Adolescents, *N* (%)	Young adults, *N* (%)	Total, *N* (%)	*p* a
1. Why do you eat fast food?				
A. Delicious taste	71 (55.9%)	29 (42%)	100 (51%)	0.188
B. Attractive advertisements	12 (9.4%)	7 (10.1%)	19 (9.7%)	
C. Diversity of fast food types	13 (10.2%)	7 (10.1%)	20 (10.2%)	
D. Convenience	25 (19.7%)	24 (34.8%)	49 (25%)	
E. Availability of fast food restaurants	6 (4.7%)	2 (2.9%)	8 (4.1%)	
2. Which issue concerns you the most regarding fast food?				
A. Restaurant's hygiene and safety	79 (62.2%)	43 (62.3%)	122 (62.2%)	0.788
B. Restaurant's location	27 (21.3%)	12 (17.4%)	39 (19.9%)	
C. Restaurant's staff	6 (4.7%)	4 (5.8%)	10 (5.1%)	
D. Price	1 (0.8%)	2 (2.9%)	3 (1.5%)	
E. Quality	11 (8.7%)	5 (7.2%)	16 (8.2%)	
F. Nutritional value	3 (2.4%)	3 (4.3%)	6 (3.1%)	
3. How do you find the taste of fast food?				
A. Excellent	109 (85.8%)	49 (71%)	158 (80.6%)	0.021
B. Acceptable	15 (11.8%)	19 (27.5%)	34 (17.3%)	
C. Bad	3 (2.4%)	1 (1.4%)	4 (2%)	
4. How do you find the price of fast food?				
A. High	17 (13.4%)	9 (13%)	26 (13.3%)	0.989
B. Acceptable	105 (82.7%)	57 (82.6%)	162 (82.7%)	
C. Low	5 (3.9%)	3 (4.3%)	8 (4.1%)	
5. How do you find the nutritional value of fast food?				
A. High	18 (14.2%)	7 (10.1%)	25 (12.8%)	0.097
B. Acceptable	50 (39.4%)	29 (42%)	79 (40.3%)	
C. Low	19 (15%)	19 (27.5%)	38 (19.4%)	
D. Don't know	40 (31.5%)	14 (20.3%)	54 (27.6%)	

a Categorical variables were expressed as numbers and percentages, and analyzed using a chi-square test.

### Fast food restaurant use

Our results revealed that the vast majority of the subjects (92.9%) purchased fast food meals from several restaurants (Table 5). However, a significantly higher rate of adolescent girls (10.2%) than young adult girls (1.4%) usually purchased fast food meals from a specific restaurant. International restaurants were preferable by participants for buying fast food compared with local restaurants (70.9% vs. 29.1%). Enjoying the delicious taste of fast food meals served by international restaurants was the major reason for preferring these restaurants by both age groups (44.9%). On the other hand, girls who preferred local restaurants had mainly two reasons: delicious taste (11.2%) and ethical issues related to ensuring that the offered foods are Halal (13.8%).

**Table 5:** Participants’ response for part 3 of the fast food questionnaire (regarding the use of fast food restaurants)

Questions asked and answer levels	Adolescents, *N* (%)	Young adults, *N* (%)	Total, *N* (%)	*p* a
1. Did you buy fast food from a specific restaurant?				
A. Yes	13 (10.2%)	1 (1.4%)	14 (7.1%)	0.023
B. No	114 (89.8%)	68 (98.6%)	182 (92.9%)	
2. From where do you usually buy fast food?				
A. Local restaurants	34 (26.8%)	23 (33.3%)	57 (29.1%)	0.334
B. International restaurants	93 (73.2%)	46 (66.7%)	139 (70.9%)	
3. If you prefer local restaurants, why?				
A. Cheaper price	3 (2.4%)	0 (0%)	3 (1.5%)	0.367
B. Ethical issues	15 (11.8%)	12 (17.4%)	27 (13.8%)	
C. Encourage national production	2 (1.6%)	3 (4.3%)	5 (2.6%)	
D. Delicious taste	14 (11%)	8 (11.6%)	22 (11.2%)	
4. If you prefer international restaurants, why?				
A. Delicious taste	57 (44.9%)	31 (44.9%)	88 (44.9%)	0.754
B. Original source of product	11 (8.7%)	4 (5.8%)	15 (7.7%)	
C. Better services	25 (19.7%)	11 (15.9%)	36 (18.4%)	

a Categorical variables were expressed as numbers and percentages, and analyzed using a chi-square test.

## Discussion

This study highlighted the high prevalence of fast food restaurant use among Saudi girls who are either adolescents or young adults. Over the past few decades, Saudi Arabians have passed through dramatic lifestyle changes. These changes have been represented in two forms: dietary patterns and sedentary lifestyles. Dietary patterns today have more energy-dense foods such as fast food and sugar-sweetened beverages at the expense of nutrient-dense foods such as fruits and vegetables, especially among adolescents and young adults (25, 26). Furthermore, sedentary lifestyles are becoming particularly prevalent among Saudi people, especially females, as most of them do not engage in physical activity of sufficient duration and frequency (27). Recently, Al-Hazzaa et al. (28) reported that about 25% of Saudi adolescent girls consumed fast foods more than three times per week, whereas 6% of them ate fast foods on a daily basis. Likewise, Alfawaz (29) investigated fast food consumption patterns among female college students in Saudi Arabia and reported that about 75% of them consumed fast foods 1–2 times weekly.

Hamburgers and French fries are the products most sold by fast food industry leaders in western countries (30). Because pork meat consumption is prohibited in Islamic society, hamburger made of pork meat is not available in the Saudi market. However, other burgers (beef or chicken) not only were available but also were the main kinds of fast food usually eaten by Saudi girls, as our results suggested. Similarly, burgers were reported among popular choices for American college students at fast food restaurants (31). As expected, carbonated soft drinks were the main beverages usually consumed with fast food meals by Saudi girls. This finding agreed with previous studies. Driskell et al. (31) found that carbonated soda has been reported to be the most frequently ordered beverage with fast food meals by female college students. Bowman (32) reported that fast food restaurants provided 25% of the carbonated soda consumed by adolescent girls. In Saudi Arabia, Al-Hazzaa et al. (28) revealed that about 60% of Saudi adolescent girls consumed sugar-sweetened drinks more than 3 days per week, and 31% of girls consumed them on a daily basis.

In our study, most girls usually ordered either small (37.2%) or medium (44.9%) portion sizes of fast food meals. A previous study found that 53% of college girls reported considering smaller portion sizes of fast food (31). However, 17.9% of the participants typically eat the large portion size. Large food portions available at fast food restaurants are a considerable concern due to their high energy content (33), for example large order of French fries (7.0 ounces) provides 610 kcalories compared with 210 kcalories in a small portion size (2.4 ounces) (34). Therefore, it has been recommended that attention should be paid to the portion size of foods and beverages offered at restaurants (35). Additionally, policy approaches and government regulations are needed to reduce energy intake from fast food as most fast food chains do not respond effectively to health authorities’ calls to reduce the portion size of their menu items (36, 37). Saudi girls often consumed fast food in the weekends and evenings. This may be due to gatherings with family and friends. Driskell et al. (31) clarified that most female college students reported typically eating fast food at least once weekly at lunch (about 60%) and dinner (about 75%), but rarely at breakfast.

Uniquely, adolescent and young adult girls agreed that restaurant hygiene and safety comprised the main issue of concern regarding fast food meals. In Saudi Arabia, there has been a steady increase in food-poisoning accidents associated with fast food restaurants, especially in the warmer climate during the summer months. Meat and chicken were reported as the main items to cause these accidents (38, 39). Several studies reported that food handlers in restaurants often had a lack of knowledge and no training regarding food hygiene and safety (40, 41). That suggests a need for education training courses targeting food handlers to increase their awareness regarding safe food-handling practices (42).

Saudi girls eat fast food primarily for enjoying the delicious taste, followed by convenience. The same primary reasons were reported among American college students with reverse order, as they were choosing to eat fast food for limited time, followed by enjoyment of the taste (31). In a national representative sample of Americans, a study revealed that taste is the most important influence on food choices. Equally important, nutritional concerns are of less relevance to most people's food choices than taste (43). Only 19.4% of participants believed that fast food has low nutritional value. In the same fashion, 35% of American college girls indicated that nutrition information influenced the choices they made regarding fast food (31). Current approaches suggest that fast food restaurants should be required to clarify nutrition information such as energy and fat content on their menu boards and on product packaging. This is important to help the consumer to make better food choices before purchasing (44). The New York City Board of Health was the first government authority to approve a calorie-labeling regulation, in 2006. This regulation requires chain restaurants’ menus to contain details of the energy content of all menu items (45). Dumanovsky and colleagues (46) assessed the impact of adding calorie labeling to menu items by fast food restaurants on the energy content of individual purchases before and after full implementation of the mentioned regulation. They found that several major chains reported significant reductions after regulation; one in six lunchtime customers used the calorie information provided, and these customers made lower-calorie choices.

Today, international fast food restaurants are located in over 90 countries worldwide, including Saudi Arabia. For example, McDonald's is now operating a chain of branches in 21 Saudi cities, with 62 branches in Riyadh alone (http://www.mcdonaldsarabia.com). The brand name of foods and beverages influences consumers’ taste perceptions and consequently their food choices (47, 48). This may explain why Saudi girls preferred international restaurants to buy fast food, as they determined enjoying the delicious taste as a main reason to choose from their menu items. On the other hand, local fast food restaurants have a huge diversity in menus and size, from restaurants with a small single branch to restaurant chains with multiple branches. For example, Kudu in Saudi Arabia has branches in 38 cities, with 50 branches in Riyadh alone (http://www.kudu.com.sa). The cultural and ethical backgrounds of consumers affect their perceptions and food choices (48). Saudi girls who preferred local restaurants exhibited concerns about ethical issues to ensure that the fast food is Halal in addition to enjoying the delicious taste.

An adequate, nutritious, and balanced diet is essential to maintain health for one's lifetime. To achieve this healthy diet, fast food consumption should be limited. Hence, nutrition-related educational interventions are important to improve the dietary habits and food choices of adolescent or young adult girls. Interestingly, current evidence emphasized that significant and beneficial changes in dietary habits have been experienced by school and college students after the implementation of interactive and effective nutrition intervention programs (49, 50). Schools and colleges are appropriate settings for contacting most adolescents and young adults continuously and in a concentrated way. Therefore, they are considered one of the best avenues for nutrition education interventions targeting these two age groups (50, 51). Several techniques of nutrition interventions were suggested as effective delivery media. These techniques include using educational lectures, using web-based education, and providing dietary supplements (50). Some nutrition interventions could be traditional lectures combined with hands-on activities that focus on selecting healthier menu options from a fast food restaurant. Other nutrition interventions can use debate lectures on nutritional treatments and cooking classes to teach students how to prepare tasty, convenient, and nutritious alternatives to fast food (49, 50). The Internet is becoming increasingly central to adolescents’ and young adults’ lifestyle patterns. Web applications and social media make it possible for health promotion initiatives to reach a large audience in a short time. Therefore, the Internet is an important vehicle to deliver messages concerning nutrition information of healthier food choices and different types of fast food meals (52). Dietary supplements can be incorporated with nutrition education programs in order to enhance health outcomes, especially among groups at nutritional risk (50).

Limitations to this study include the relatively small sample size and cross-sectional design. However, our study has a number of strengths. This is the first attempt, to our knowledge, to investigate the trends of fast food consumption among adolescent and young adult Saudi girls in one study under the same procedure and by using the same tools. Furthermore, using a face-to-face interview questionnaire instead of a self-reported questionnaire helps to clarify misunderstanding, enhance the response rate, and reduce possible bias.

## Conclusion

In summary, fast food has become an important component of the dietary pattern for Saudi girls, whether adolescents or young adults, and their fast food eating is likely to continue and rise. The growing widespread use of fast food among adolescents and young adults is of concern due to the high fat and energy intake, which may cause obesity and subsequently obesity-related chronic diseases. Community-based nutrition education interventions targeting the eating behaviors of adolescent and young adult girls are urgently needed. These interventions should incorporate specific components that address the trends of fast food consumption of these age groups. It is more effective to target issues related to the preparation of tasty, convenient, and nutritious alternatives to fast food and the selection of healthier menu options from a fast food restaurant rather than to solely target the unhealthiness of fast food. Portion size selection and frequency of fast food eating might also be targeted by nutrition education campaigns that are concerned about controlling daily intake from fat and calories. Also, it is advised to make adolescents and young adults aware of the importance of making healthier food choices composed of nutrient-dense foods such as fruit and vegetables on eating occasions other than those at fast food restaurants. Finally, government legislation is needed to regulate the marketing of fast food and to eliminate fast food from schools and colleges.
